# Supplementary material for: 𝒫𝒯-symmetric interference transistor
Source: Sci Rep. 2018 Oct 25;8:15780. doi: 10.1038/s41598-018-34132-0 (PMC6202334; doi:10.1038/s41598-018-34132-0)
Supplement: Supplementary file 1 — Supplementary information [file 41598_2018_34132_MOESM1_ESM.pdf]

$\mathcal{PT}$-symmetric interference transistor (Supplementary information)

Alexander A. Gorbatsevich[1,2,*], Gennadiy Ya. Krasnikov[2], and Nikolay M. Shubin[1,2,3]

[1]P.N. Lebedev Physical Institute of the Russian Academy of Sciences, Division of solid state physics, Moscow, 119991, Russia.
[2]JSC Research Institute of Molecular Electronics and "Mikron" Plant, Zelenograd, Moscow, 124460, Russia.
[3]National Research University of Electronic Technology, Department of quantum physics and nanoelectronics, Zelenograd, Moscow, 124498, Russia.
[*]aagor137@mail.ru

1 $\mathcal{PT}$-symmetry and exceptional points

From the very first announcement by Bender and Boettcher [1] that some special non-Hermitian Hamiltonians can posses real spectrum, which can spontaneously transform to complex ($\mathcal{PT}$-symmetry breaking), there was a huge interest to this field as it reopens the novel physics of non-Hermitian Hamiltonians and fills it by a realistic physical sense. The Hamiltonians studied by Bender and co-workers are so-called $\mathcal{PT}$-symmetric Hamiltonians (operators), which might be non-Hermitianm but are invariant under simultaneous space inversion ($\mathcal{P}$) and time reversal ($\mathcal{T}$). Since then there were introduced a lot of $\mathcal{PT}$-symmetric models describing various physical phenomena in the fields of quantum mechanics, optics, condensed matter and other. Up to now there are some exhaustive reviews on the formalism of $\mathcal{PT}$-symmetry (e.g. [2, 3, 4]). Here we briefly state some basic concepts of this formalism to clarify the notation and general properties of $\mathcal{PT}$-symmetry and $\mathcal{PT}$-symmetry breaking phenomenon.

Consider the simplest 2×2 $\mathcal{PT}$-symmetric Hamiltonian as an illustrative example:

$$\hat{H}_{\mathcal{PT}} = \begin{pmatrix} i\Gamma & \tau \\ \tau^* & -i\Gamma \end{pmatrix}, \tag{Suppl. 1}$$

where $\Gamma > 0$ and $\tau \in \mathbb{C}$ are some parameters. One can understand (Suppl. 1) as a Hamiltonian of a dimer with complex on-site "potentials" $\pm i\Gamma$ and intersite hopping τ. Hamiltonian $\hat{H}_{\mathcal{PT}}$ is clearly non-Hermitian, but it is invariant under the following transformation:

$$\mathcal{PT}\hat{H}_{\mathcal{PT}}(\mathcal{PT})^{-1} = \mathcal{PT}\hat{H}_{\mathcal{PT}}\mathcal{PT} = \hat{H}_{\mathcal{PT}}, \tag{Suppl. 2}$$

where $\mathcal{P} = \begin{pmatrix} 0 & 1 \\ 1 & 0 \end{pmatrix}$ is the spatial inversion for this two state system (in the site basis) and the time reversal operation $\mathcal{T}$ is just a complex conjugation. The last equality in (Suppl. 2) is understood as the LHS and RHS acts equally on any vector $\mathbf{v} \in \mathbb{C}^2$. Generally, spatial inversion operator might be different to this one (e.g. in another basis), but it must satisfy the condition $\mathcal{P} = \mathcal{P}^{-1}$ and commute with $\mathcal{T}$. From Eq. (Suppl. 1) One can easily calculate eigenvalues and eigenvectors of $\hat{H}_{\mathcal{PT}}$:

$$\begin{aligned} |1\rangle &= \left(\frac{i\Gamma + \sqrt{|\tau|^2 - \Gamma^2}}{\tau^*}, 1\right)^\top, \quad E_1 = \sqrt{|\tau|^2 - \Gamma^2}; \\ |2\rangle &= \left(\frac{i\Gamma - \sqrt{|\tau|^2 - \Gamma^2}}{\tau^*}, 1\right)^\top, \quad E_2 = -\sqrt{|\tau|^2 - \Gamma^2}. \end{aligned} \tag{Suppl. 3}$$

It turns out that non-Hermitian Hamiltonian $\hat{H}_{\mathcal{PT}}$ has an entirely real spectrum for $|\tau| > \Gamma$ and its eigenvalues are orthogonal: $\langle 1|2\rangle = \langle 2|1\rangle = 0$. This is the $\mathcal{PT}$-symmetric phase. Spontaneously, for $|\tau| = \Gamma$ we have the $\mathcal{PT}$-symmetry breaking phenomenon, i.e. the situation, where both eigenvectors and eigenvalues coalesce. In this case Hamiltonian (Suppl. 1) becomes nondiagonalizable and it can be written only as a Jordan block. This is called exceptional point (EP) in the spectral theory of linear operators [5] and it cannot take place for a Hermitian operator. For $|\tau| > \Gamma$ we again have orthogonal eigenstates, but with purely imaginary eigenvalues, which are complex conjugates of each other. This is $\mathcal{PT}$-symmetry broken phase.

In the present paper we use the formalism of $\mathcal{PT}$-symmetry and $\mathcal{PT}$-symmetry breaking to describe perfect transmission peaks and the phenomenon of resonance coalescence (in the sense of Ref. [6]).

2 Microscopic parameters

2.1 Couplings to the leads

Tunneling coupling to the left (right) lead of an N site system within tight-binding approximation can be described by a vector $\mathbf{u}^{site}_{L(R)} \in \mathbb{C}^N$ composed of hoping integrals from each site of the system to the left (right) lead [6], which define the coupling strength to the corresponding lead. Thus, we denote $\mathbf{u}^{site}_{L(R)}$ as a lead coupling vector in the basis of on-site localized states. Transformation to any other orthonormal basis is done by some unitary matrix U:

$$\mathbf{u}^{new}_{L(R)} = U^\dagger \mathbf{u}^{site}_{L(R)}. \qquad \text{(Suppl. 4)}$$

Matrix U is composed of column-vectors of new basis, thus, from Eq. (Suppl. 4) one can see that elements of the transformed vector $\mathbf{u}^{new}_{L(R)}$ are just scalar products of $\mathbf{u}^{site}_{L(R)}$ and basis vectors (projections for orthonormal basis). Therefore, transforming basis to the eigenstates of the bare Hamiltonian of the system we get a $\mathbf{u}^{eig}_{L(R)}$ composed of scalar products of $\mathbf{u}^{site}_{L(R)}$ with all the eigenstates. In particular, if the leads interact only with single site each, i.e. $\mathbf{u}^{site}_{L(R)}$ has only one nonzero element each, the scalar products will result in just components of new basis vectors corresponding to the contacting sites (multiplied by a factor governing the coupling strength).

Consider a two-dimensional subspace $\mathcal{H}_{12}$ of the total N-dimensional Hilbert space $\mathcal{H}$, which is spanned by symmetric $|s\rangle$ and anti-symmetric $|a\rangle$ degenerate states.[1] Components of the vector $\mathbf{u}^{eig}_{L(R)}$ belonging to the subspace $\mathcal{H}_{12}$ are the scalar products of initial coupling vector in the site basis with the symmetric and anti-symmetric states. This two components are arranged into a vector $\mathbf{u}_{L(R)}$, which is used in the main text. If the leads are attached in a symmetrical way, i.e. the left lead interacts with the site n in the same way as the right lead interacts with the site $\sigma_{LR} n$, then, obviously, scalar products of $\mathbf{u}^{site}_L$ with $|s\rangle$ and $\mathbf{u}^{site}_R$ with $|s\rangle$ will be the same. On the other hand, dot products of $\mathbf{u}^{site}_L$ with $|a\rangle$ and $\mathbf{u}^{site}_R$ with $|a\rangle$ will have equal absolute values, but opposite signs.

2.2 Perturbation induced shifts of degenerate states energies

Small shifts of degenerate states energies induced by some perturbation $\hat{V}$ can be dealt with by a perturbation theory for degenerate states [7]. To get first order corrections to the energy one should solve a secular equation, which for two degenerate states $|s\rangle$ and $|a\rangle$ can be done easily:

$$\Delta E^{(1)}_{s,a} = \frac{\langle s|\hat{V}|s\rangle + \langle a|\hat{V}|a\rangle}{2} \pm \frac{1}{2}\sqrt{\left(\langle s|\hat{V}|s\rangle - \langle a|\hat{V}|a\rangle\right)^2 + 4\left|\langle s|\hat{V}|a\rangle\right|^2}. \qquad \text{(Suppl. 5)}$$

Here $\langle x|\hat{V}|y\rangle$ is the matrix element of the perturbation operator between some states $|x\rangle$ and $|y\rangle$. Corrections (Suppl. 5) are linear in perturbation strength and in some cases it may turn out that $\Delta E^{(1)}_s = \Delta E^{(1)}_a$, i.e. perturbation does not remove the degeneracy in the first order. This situation, for example, may takes place for disjoint diradicals. Indeed, if the perturbation influences only on-site energies (i.e. it is diagonal in the site basis), then matrix elements $\langle s|\hat{V}|a\rangle$ and $\langle a|\hat{V}|s\rangle$ turn exactly to zero for disjoint diradicals, as states $|s\rangle$ and $|a\rangle$ share no common atomic orbitals. Hence, according to Eq. (Suppl. 5), if diagonal matrix elements $\langle s|\hat{V}|s\rangle$ and $\langle a|\hat{V}|a\rangle$ are equal, then first order corrections to the energy become equal as well. In this case we should take into

[1] Symmetry is meant to be with respect to the mirror symmetry σ_{LR} mapping left and right leads into each other (see main text).

account not only degenerate states, but all other states with different energies. This can be done by renormalization of perturbation matrix elements [7]:

$$\langle x|\hat{V}|y\rangle \mapsto \langle x|\hat{V}|y\rangle + \sum_{n;n\neq s,a} \frac{\langle x|\hat{V}|n\rangle \langle n|\hat{V}|y\rangle}{E_x^0 - E_n^0}, \qquad \text{(Suppl. 6)}$$

where x and y take values s or a and E_n^0 is the energy of the state $|n\rangle$ without perturbation. This procedure involves the second order perturbation theory corrections and, thus, resulting energy shifts become non-linear (quadratic) with the perturbation strength.

3 Switching properties of the PT-symmetric interference transistor

The ballistic current through a quantum conductor sandwiched between two leads with applied voltages V_L and V_R can be calculated by a standard formula [8, 9, 10]:

$$I = \frac{e}{h} \int T(\omega) \left[f_L(\omega) - f_R(\omega)\right] d\omega, \qquad \text{(Suppl. 7)}$$

where $T(\omega)$ is the transmission coefficient and $f_{L(R)}(\omega) = \{1 + \exp[(\omega + eV_{L(R)})/kT]\}^{-1}$ is the Fermi-Dirac distribution function in the left (right) lead and the energy ω is measured from the Fermi energy of unbiased leads.

The difference $\Delta f = f_L(\omega) - f_R(\omega)$ defines an effective integral domain in (Suppl. 7). Moreover, for small bias voltages (compared to kT/e) between the left and the right lead, this difference of distribution functions also introduces a scaling factor, because its maximum value becomes less than 1. Indeed, if the voltage V_{LR} between the leads us applied in a symmetric form: $V_L = -V_{LR}/2$ and $V_R = V_{LR}/2$, then

$$\Delta f(\omega) = f_L(\omega) - f_R(\omega) = \frac{\sinh \frac{eV_{LR}}{2kT}}{\cosh \frac{eV_{LR}}{2kT} + \cosh \frac{\omega}{kT}}. \qquad \text{(Suppl. 8)}$$

From Eq. (Suppl. 8) one can see that $\Delta f(\omega)$ reaches its maximum value $\Delta f_{max} = \tanh \frac{eV_{LR}}{4kT}$ at $\omega = 0$ (energy is measured from the Fermi level of the unbiased leads) and its half width at half maximum (HWHM) is $\Delta f_{HWHM} = kT \operatorname{arcosh}\left(2 + \cosh \frac{eV_{LR}}{2kT}\right)$. For small bias voltages it is easy to estimate $\Delta f_{max} \approx \frac{eV_{LR}}{4kT}$ and $\Delta f_{HWHM} \approx kT \operatorname{arcosh} 3 \approx 1.76kT$. Thus, in this case the width of the effective integral domain in (Suppl. 7) is governed mainly by kT and the effective scale factor, proportional to $\Delta f_{max} = \tanh \frac{eV_{LR}}{4kT} \approx \frac{eV_{LR}}{4kT}$, is introduced. For transmission resonances width, much smaller than kT, we can estimate the current as

$$I \approx \frac{e}{h} \times \Delta f(\varepsilon_0) \times \int_{-\infty}^{\infty} T(\omega) d\omega, \qquad \text{(Suppl. 9)}$$

where energy ε_0 defines the position of the transmission resonance. If ε_0 is close to the Fermi level of the unbiased lead (i.e. $\varepsilon_0 \approx 0$), then, using Eq. (Suppl. 8), $\Delta f(\varepsilon_0)$ can be estimated as $\Delta f(\varepsilon_0) \approx \tanh \frac{eV_{LR}}{4kT}$. Therefore, in this case, according to Eq. (Suppl. 9) there are two regimes of the current flow: the ohmic regime with $V_{LR} < kT/e$ and $\tanh \frac{eV_{LR}}{4kT} \approx \frac{eV_{LR}}{4kT}$, when the current linearly grows with increasing V_{LR}, and the saturation regime with $V_{LR} \gg kT/e$ and $\tanh \frac{eV_{LR}}{4kT} \approx 1$, when the current almost does not change with varying V_{LR}.

Therefore, the ballistic current through the considered microscopic model calculated via Eq. (Suppl. 7) can be calculated by integration of the transmission coefficient from Eq. (9) of the main text by standard methods (e.g. applying Cauchy residue theorem):

$$I \approx \frac{e\Gamma}{\hbar} \times \tanh \frac{eV_{LR}}{4kT} \times \frac{\left(\gamma_s^2 + \gamma_a^2\right) \left[\delta^2 \left(k_a - k_s\right)^2 + 4\Gamma^2 \left(\gamma_a^2 - \gamma_s^2\right)^2\right]}{\delta^2 \left(k_a - k_s\right)^2 + 4\Gamma^2 \left(\gamma_s^2 + \gamma_a^2\right)^2}. \qquad \text{(Suppl. 10)}$$

Parameters $\gamma_{a,s}$, $k_{a,s}$ and δ are specified in the main text. In order to investigate the current switching by varying δ we can estimate I_{on} and I_{off} currents and their ratio. The current (Suppl. 10) reaches its minimum value at $\delta = 0$ and it monotonically grows to its asymptotic maximum as δ goes to infinity. Hence, we can set $\delta = 0$

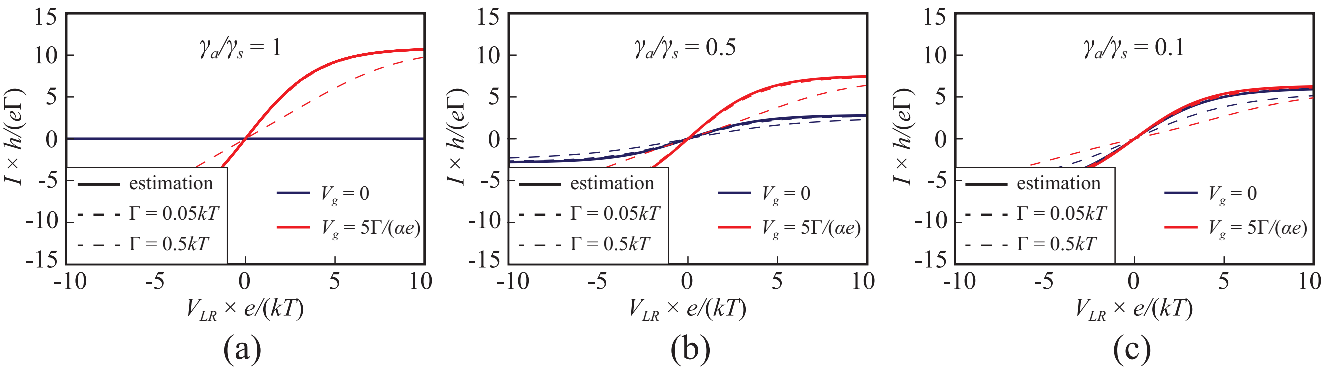

Suppl. Figure 1: I-V characteristics of the PT-symmetric interference transistor based on a system with $k_a = -k_s = 1$, $\gamma_s = 1$, and $\gamma_a = 1$ (a), $\gamma_a = 0.5$ (b), and $\gamma_a = 0.1$ (c). Blue lines corresponds to the "off" state ($V_g = 0$) and red line to the "on" state with $V_g > 0$. Switching properties degrade as γ_a goes away from γ_s. Thick solid lines show the estimation of the current by Eq. (Suppl. 7) and different dashed lines corresponds to resonances with different width (compared to kT). Obviously, the thinner resonances are the more accurate estimation by Eq. (Suppl. 7) becomes. Switching between "on" and "off" states becomes negligibly as γ_s and γ_a difference becomes significant (c).

to estimate the minimum current (i.e. I_{off}) and some $\delta = \delta_0 > 0$ to estimate I_{on}. For instance we can take $\delta_0 = 4\gamma_s\gamma_a\Gamma|k_a - k_s|^{-1}$, which corresponds to the coalescence of resonances. So, we get:

$$I_{on} \approx \frac{e\Gamma}{\hbar} \times \tanh\frac{eV_{LR}}{4kT} \times \frac{\left(\gamma_s^2+\gamma_a^2\right)^3}{\gamma_a^4+\gamma_s^4+6\gamma_a^2\gamma_s^2},$$
$$I_{off} \approx \frac{e\Gamma}{\hbar} \times \tanh\frac{eV_{LR}}{4kT} \times \frac{\left(\gamma_s^2-\gamma_a^2\right)^2}{\gamma_s^2+\gamma_a^2}. \qquad \text{(Suppl. 11)}$$

Therefore, the on/off current ratio appears to be $I_{on}/I_{off} \propto (\gamma_s - \gamma_a)^{-2}$. Thus, it can be infinitely (!) large as γ_s goes to γ_a (without taking into account contribution to the I_{off} from the off-resonant tunnelling through distant energy levels). As an illustration of the quantum interference switch operation there are numerically calculated [via. Eq. (Suppl. 7)] I-V characteristics in Fig. 1 and current vs. control (gate) voltage plots in Fig. 2.

One of the main characteristics of an electronic switch is its transconductance (i.e. the current change per unit gate voltage increment). In our system we suppose that the gate controls the detuning parameter δ via some electrostatical lever arm $0 < \alpha < 1$:

$$\delta = \alpha e V_g, \qquad \text{(Suppl. 12)}$$

where V_g is the control (gate) voltage. From Eq. (Suppl. 10) we can conclude, that the highest transconductance ($g = \frac{\partial I}{\partial V_g}$) is achieved at $V_g = 2\Gamma\frac{\gamma_s^2+\gamma_a^2}{\sqrt{3}\alpha e|k_a-k_s|}$:

$$g_{max} = \frac{e^2}{h} \times \tanh\frac{eV_{LR}}{4kT} \times \frac{3\sqrt{3}\alpha\gamma_s^2\gamma_a^2\,|k_a-k_s|\,\pi}{2\left(\gamma_s^2+\gamma_a^2\right)^2}. \qquad \text{(Suppl. 13)}$$

The possible maximum value for it corresponds to the ideal case ($\alpha \to 1$, $\gamma_a/\gamma_s \to 1$, and $k_a = -k_s = 1$): $g_{max} \to \frac{e^2}{h} \times \tanh\frac{eV_{LR}}{4kT} \times \frac{3\sqrt{3}\pi}{4}$, which in the saturation regime approaches the value $\frac{e^2}{h} \times \frac{3\sqrt{3}\pi}{4} \approx 160\mu\text{A/V}$. Alternatively, we can estimate the average transconductance as $g_{avg} = (I_{on} - I_{off})/\Delta V_g$, where ΔV_g is the range of the gate voltage needed to turn the current from I_{off} to I_{on}: $\Delta V_g = \frac{4\gamma_s\gamma_a\Gamma}{\alpha e|k_a-k_s|}$. Hence, using Eq. (Suppl. 11) we get that

$$g_{avg} = \frac{e^2}{h} \times \tanh\frac{eV_{LR}}{4kT} \times \frac{8\pi\alpha\gamma_s^3\gamma_a^3\,|k_a-k_s|}{\left(\gamma_s^2+\gamma_a^2\right)\left(\gamma_s^4+\gamma_a^4+6\gamma_s^2\gamma_a^2\right)} \qquad \text{(Suppl. 14)}$$

and the optimal case is again $\alpha \to 1$, $\gamma_a/\gamma_s \to 1$, and $k_a = -k_s = 1$, which results in $g_{avg} = \frac{e^2\pi}{h} \times \tanh\frac{eV_{LR}}{4kT}$. In the saturation regime it approaches the value $\frac{e^2\pi}{h} \approx 120\mu\text{A/V}$.

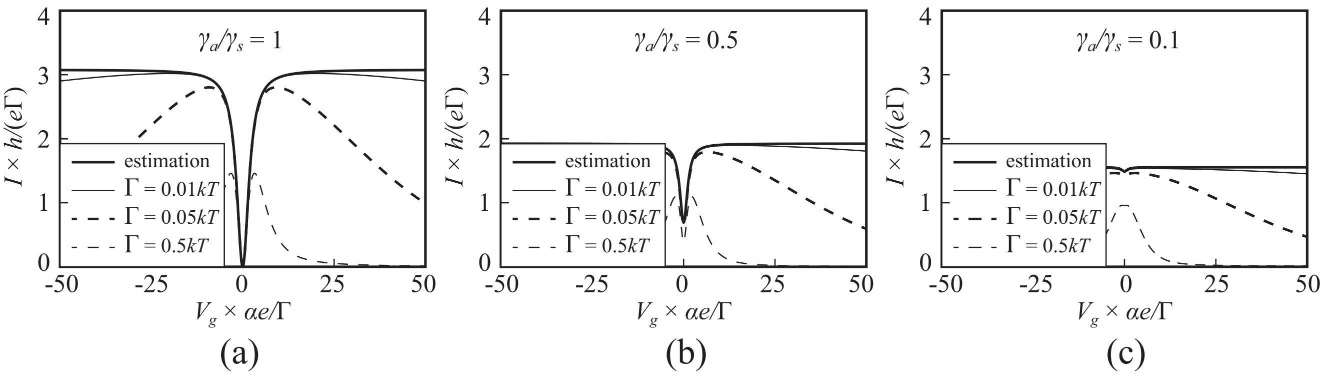

Suppl. Figure 2: Current vs. gate voltage plots of the PT-symmetric interference transistor based on a system with $k_a = -k_s = 1$, $\gamma_s = 1$, and $\gamma_a = 1$ (a), $\gamma_a = 0.5$ (b), and $\gamma_a = 0.1$ (c) at $V_{LR} = kT/e$. Switching properties degrade as γ_a goes away from γ_s. Thick solid lines show the estimation of the current by Eq. (Suppl. 10) and different dashed lines corresponds to resonances with different width (compared to kT). Violation from estimation by Eq. (Suppl. 7) here is due to the partial resonance shift out of the effective integral range in Eq. (Suppl. 7). The wider the resonance is, the stronger this effect becomes.

Another important small signal parameter of any transistor (or switch) is the gain K of the simplest amplifier composed of the transistor and some load resistance R_{load}. According to Ref. [11], gain in this case is given by the following expression:

$$K = \frac{g}{g_0 + 1/R_{load}}, \tag{Suppl. 15}$$

where g is transconductance and $g_0 = \frac{\partial I}{\partial V_{LR}}$ is output conductance. From Eq. (Suppl. 10) one can see that in the ohmic regime g_0 is given by

$$g_0 \approx \frac{e^2}{h} \times \frac{\pi\Gamma}{2kT} \times \frac{\left(\gamma_s^2 + \gamma_a^2\right)\left[\delta^2\left(k_a - k_s\right)^2 + 4\Gamma^2\left(\gamma_a^2 - \gamma_s^2\right)^2\right]}{\delta^2\left(k_a - k_s\right)^2 + 4\Gamma^2\left(\gamma_s^2 + \gamma_a^2\right)^2}, \tag{Suppl. 16}$$

and in the saturation regime $g_0 \approx 0$. Hence, formally, the gain can be made arbitrary high by adjusting appropriate load in the saturation regime. On the other hand, however, in the ohmic regime the gain is bounded [11] to $K_{max} = g/g_0$, which for ideal case ($k_a = -k_s = 1$, $\alpha \to 1$, and $\gamma_s = \gamma_a = 1$) reduces to:

$$K_{max} = \frac{g}{g_0} = \frac{V_{LR}}{V_g} \times \frac{8}{4 + \left(\frac{eV_g}{\Gamma}\right)^2}. \tag{Suppl. 17}$$

4 Transmission coefficient of diradical molecules models

4.1 Model of non-disjoint diradical (trimethylenemethane)

Consider the structure shown in Suppl. Fig. 3a; it schematically corresponds to a Hückel tight-binding model of an electronic subsystem composed of atomic p-orbitals in trimethylenemethane molecule. Bare Hamiltonian of this system is

$$\hat{H}_0 = \begin{pmatrix} \varepsilon_0 & 0 & \tau_1 & 0 \\ 0 & \varepsilon_1 & \tau & 0 \\ \tau_1 & \tau & \varepsilon_1 & \tau_1 \\ 0 & 0 & \tau_1 & \varepsilon_0 \end{pmatrix}. \tag{Suppl. 18}$$

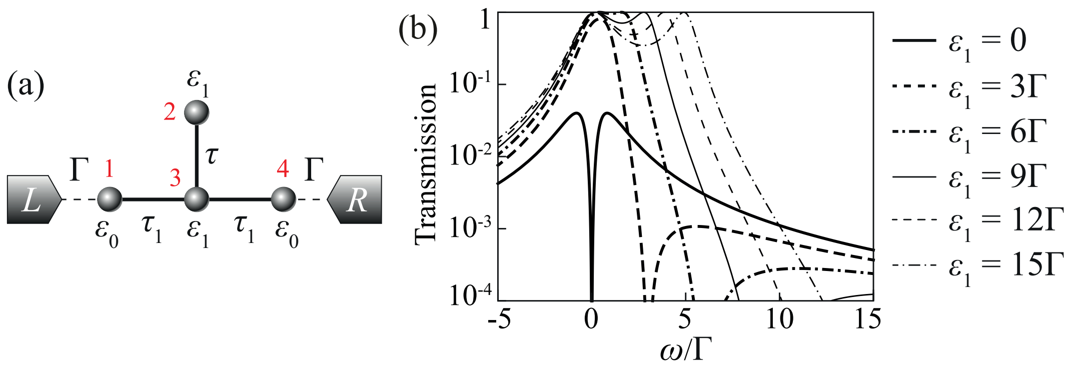

Suppl. Figure 3: Model of a non-disjoint diradical. (a) Schematic view of a Hückel tight-binding structure of a trimethylenemethane molecule, which has two carbon atoms (with on-site p-orbital energies ε_1) biased by an external gate. (b) Transmission coefficient of this system with different values of ε_1 and parameters $\varepsilon_0 = 0$, $\tau = 100\Gamma$ and $\tau_1 = 50\Gamma$.

Here $\varepsilon_{0,1}$ are on-site potentials and τ and τ_1 are double and single bond hopping integrals. First of all we can calculate from symmetric $|s\rangle$ and anti-symmetric $|a\rangle$ degenerate eigenstates with energies $\omega = \varepsilon_0$ at $\varepsilon_1 = \varepsilon_0$:

$$\begin{aligned} |s\rangle &= \left(\frac{\tau}{\sqrt{2\left(2\tau_1^2+\tau^2\right)}}, -\frac{\sqrt{2}\tau_1}{\sqrt{2\tau_1^2+\tau^2}}, 0, \frac{\tau}{\sqrt{2\left(2\tau_1^2+\tau^2\right)}} \right)^\top, \\ |a\rangle &= \left(\frac{1}{\sqrt{2}}, 0, 0, -\frac{1}{\sqrt{2}} \right)^\top. \end{aligned} \tag{Suppl. 19}$$

Besides these degenerate states molecule has 2 more molecular orbitals, which form some distant (from $\omega = \varepsilon_0$) resonances in the transmission spectrum, which are not considered by our microscopic model, but are essentially taken into account by the following calculations as we use the full 4×4 Hamiltonian $\hat{H}_0$ from Eq. (Suppl. 18). As the leads are connected to the first and to the fourth sites (Suppl. Fig. 3a), couplings to them can be arranged into the following vectors:

$$\begin{aligned} \mathbf{u}_L &= \sqrt{\Gamma} \begin{pmatrix} \frac{\tau}{\sqrt{2\left(2\tau_1^2+\tau^2\right)}} \\ 1/\sqrt{2} \end{pmatrix}, \\ \mathbf{u}_R &= \sqrt{\Gamma} \begin{pmatrix} \frac{\tau}{\sqrt{2\left(2\tau_1^2+\tau^2\right)}} \\ -1/\sqrt{2} \end{pmatrix}. \end{aligned} \tag{Suppl. 20}$$

Thus, parameters $\gamma_{s,a}$ are

$$\gamma_s = \frac{\tau}{\sqrt{2\left(2\tau_1^2+\tau^2\right)}}, \quad \gamma_a = 1/\sqrt{2}. \tag{Suppl. 21}$$

The perturbation, which detune the system from the degenerate state ($\varepsilon_1 = \varepsilon_0$) is given in the basis of atomic orbitals in the following diagonal form:

$$\hat{V} = \begin{pmatrix} 0 & 0 & 0 & 0 \\ 0 & \varepsilon_1 - \varepsilon_0 & 0 & 0 \\ 0 & 0 & \varepsilon_1 - \varepsilon_0 & 0 \\ 0 & 0 & 0 & 0 \end{pmatrix}. \tag{Suppl. 22}$$

Using Eq. (Suppl. 5) we can estimate energy shift of states $|s\rangle$ and $|a\rangle$ induced by the perturbation (Suppl. 22). Hence, parameters $k_{a,s}$ are

$$k_s = \frac{2\tau_1^2}{2\tau_1^2+\tau^2}, \quad k_a = 0. \tag{Suppl. 23}$$

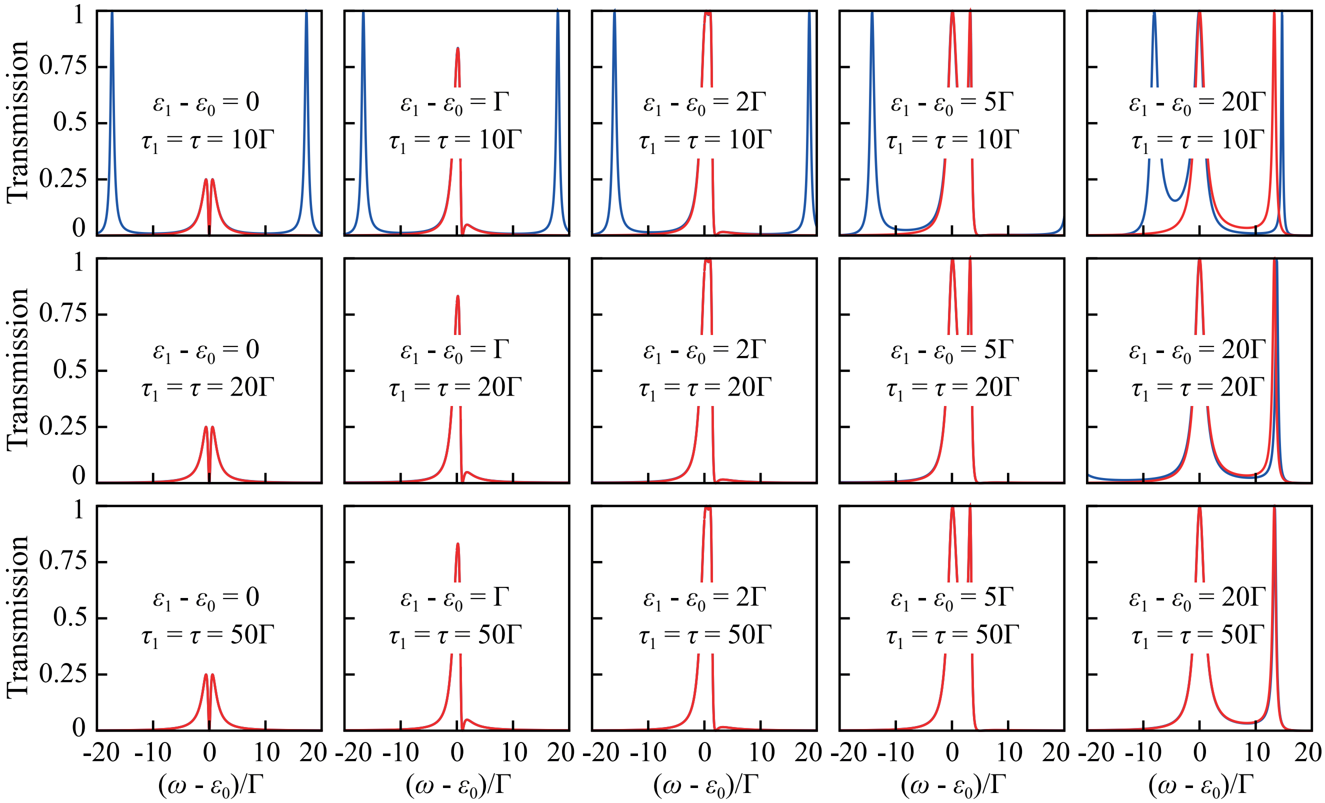

Suppl. Figure 4: Exact (blue lines) and estimated (red lines) transmission coefficient of a tight-binding Hückel model of trimethylenemethane. As couplings to the leads Γ becomes smaller compared to intermolecular hopping integrals τ and τ_1, the estimation of the transmission coefficient within our microscopic model becomes more and more accurate.

Finally we can calculate exact expression for the transmission coefficient within the wide-band limit in the form of Eq. (1) of the main text:

$$T_{ND} = \frac{4\Gamma^2\tau_1^4\tilde{\omega}^2}{Q_{ND} + 4\Gamma^2\tau_1^4\tilde{\omega}^2}, \qquad \text{(Suppl. 24)}$$

with

$$Q_{ND} = \tilde{\omega}^4 - 2\tilde{\varepsilon}_1\tilde{\omega}^3 + \left(\tilde{\varepsilon}_1^2 + \Gamma^2 - \tau^2 - 2\tau_1^2\right)\tilde{\omega}^2 + 2\tilde{\varepsilon}_1\left(\tau_1^2 - \Gamma^2\right)\tilde{\omega} + \Gamma^2\left(\tilde{\varepsilon}_1^2 - \tau^2\right), \qquad \text{(Suppl. 25)}$$

where $\tilde{\omega} = \omega - \varepsilon_0$ and $\tilde{\varepsilon}_1 = \varepsilon_1 - \varepsilon_0$. Figure 4 shows the correspondence between exact transmission coefficient expression and estimation via our microscopic model with parameters $\gamma_{s,a}$ and $k_{s,a}$ obtained above. All energies are measured in units of Γ.

4.2 Model of disjoint diradical (divinylcyclobutadiene)

Consider the structure shown in Suppl. Fig. 5a, which schematically corresponds to a Hückel tight-binding model of an electronic subsystem composed of atomic p-orbitals in divinylcyclobutadiene molecule. The bare Hamiltonian $\hat{H}_0$ of this system is following:

$$\hat{H}_0 = \begin{pmatrix} \varepsilon_1 & \tau & \tau & 0 & 0 & 0 & 0 & 0 \\ \tau & \varepsilon_1 & 0 & \tau & 0 & 0 & 0 & 0 \\ \tau & 0 & \varepsilon_0 & \tau & \tau & 0 & 0 & 0 \\ 0 & \tau & \tau & \varepsilon_0 & 0 & \tau & 0 & 0 \\ 0 & 0 & \tau & 0 & \varepsilon_0 & 0 & \tau & 0 \\ 0 & 0 & 0 & \tau & 0 & \varepsilon_0 & 0 & \tau \\ 0 & 0 & 0 & 0 & \tau & 0 & \varepsilon_0 & 0 \\ 0 & 0 & 0 & 0 & 0 & \tau & 0 & \varepsilon_0 \end{pmatrix}. \qquad \text{(Suppl. 26)}$$

Here again $\varepsilon_{0,1}$ are on-site potentials and τ is the hopping integral of each bond, which are taken to be equal. The Hamiltonian $\hat{H}_0$ in this expression is written in the basis of localized states, which are labeled in order from left to right and from up to down (see Fig. 5a). Degenerate eigenstates at energy $\omega = \varepsilon_0$ appear if we set $\varepsilon_1 = \varepsilon_0$. In the basis of localized states symmetric $|s\rangle$ and anti-symmetric states $|a\rangle$ with respect to the mirror symmetry[2] are following

$$\begin{aligned} |s\rangle &= \frac{1}{\sqrt{6}}\left(1, 1, -1, -1, 0, 0, 1, 1\right)^\top, \\ |a\rangle &= \frac{1}{\sqrt{6}}\left(1, -1, -1, 1, 0, 0, 1, -1\right)^\top. \end{aligned} \qquad \text{(Suppl. 27)}$$

Besides these degenerate states molecule has 6 more molecular orbitals, which form some distant (from $\omega = \varepsilon_0$) resonances in the transmission spectrum, which are not considered by our microscopic model, but are essentially taken into account by the following calculations as we use the full 8×8 Hamiltonian $\hat{H}_0$ from Eq. (Suppl. 26). Leads are attached to the 7-th and to the 8-th sites and hence the coupling strength of symmetric and anti-symmetric states to them can be described by following vectors:

$$\begin{aligned} \mathbf{u}_L &= \sqrt{\frac{\Gamma}{6}}\begin{pmatrix} 1 \\ 1 \end{pmatrix}, \\ \mathbf{u}_R &= \sqrt{\frac{\Gamma}{6}}\begin{pmatrix} 1 \\ -1 \end{pmatrix}. \end{aligned} \qquad \text{(Suppl. 28)}$$

Comparing Eq. (Suppl. 28) with general expression (5) of the main text, one can easily see that $\gamma_s = \gamma_a = 6^{-\frac{1}{2}}$ for this structure. Hence, from Eq. (9) of the main text transmission must turn exactly to zero as $\varepsilon_1 = \varepsilon_0$, but from Fig. 5b one can see that in this case transmission lowers, but remains finite. This is due to contribution of the distant resonances, which are not taken into account while deriving Eq. (9) of the main text.

[2] Symmetry σ_{LR}, which maps the leads into each other. It acts on the sites of the system as the permutation $\sigma_{LR} = \begin{pmatrix} 1 & 2 & 3 & 4 & 5 & 6 & 7 & 8 \\ 2 & 1 & 4 & 3 & 6 & 5 & 8 & 7 \end{pmatrix}$

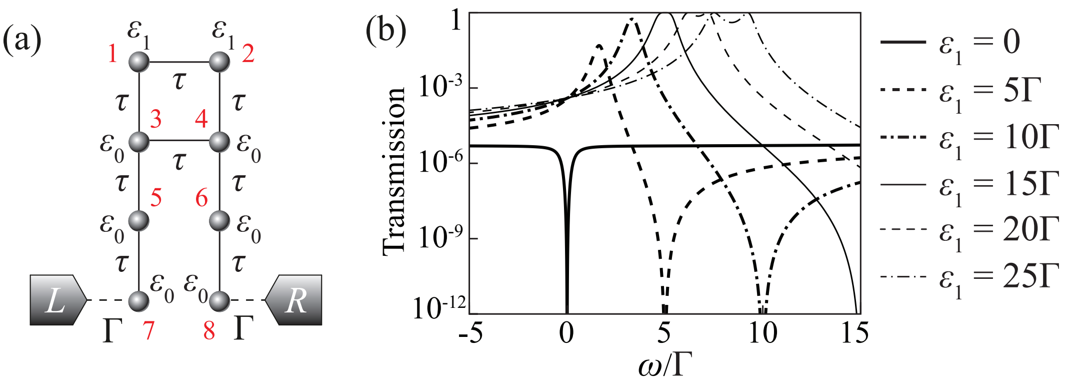

Suppl. Figure 5: Model of a disjoint diradical. (a) Schematic view of a Hückel tight-binding structure of a divinylcyclobutadiene molecule, which has two carbon atoms (with on-site p-orbital energies ε_1) biased by an external gate. (b) Transmission coefficient of this system with different values of ε_1 and parameters $\varepsilon_0 = 0$ and $\tau = 100\Gamma$. At $\varepsilon_1 = \varepsilon_0 = 0$ (thick solid line) the transmission turns to zero only in the vicinity of $\omega = \varepsilon_0 = 0$ due to the presence of a "background" transmission arising from distant (in energy) resonances.

Perturbation operator in this case is

$$\hat{V} = \begin{pmatrix} \varepsilon_1 - \varepsilon_0 & 0 & 0 & 0 & 0 & 0 & 0 & 0 \\ 0 & \varepsilon_1 - \varepsilon_0 & 0 & 0 & 0 & 0 & 0 & 0 \\ 0 & 0 & 0 & 0 & 0 & 0 & 0 & 0 \\ 0 & 0 & 0 & 0 & 0 & 0 & 0 & 0 \\ 0 & 0 & 0 & 0 & 0 & 0 & 0 & 0 \\ 0 & 0 & 0 & 0 & 0 & 0 & 0 & 0 \\ 0 & 0 & 0 & 0 & 0 & 0 & 0 & 0 \\ 0 & 0 & 0 & 0 & 0 & 0 & 0 & 0 \end{pmatrix}. \qquad \text{(Suppl. 29)}$$

Using this expression in Eq. (Suppl. 5), one can conclude that first order corrections to the energies of the symmetric and anti-symmetric states are equal: $\Delta E^{(1)}_{s,a} = \frac{1}{3}(\varepsilon_1 - \varepsilon_0)$. Therefore, we need to take into account further corrections, following Eq. (Suppl. 6). Thus, we get non-linear energy shifts and, consequently, parameters $k_{s,a}$ depending on the detuning:

$$k_s = \frac{1}{3} + \frac{4}{27}(\varepsilon_1 - \varepsilon_0), \quad k_a = \frac{1}{3} - \frac{4}{27}(\varepsilon_1 - \varepsilon_0). \qquad \text{(Suppl. 30)}$$

The exact expression for the transmission coefficient of this structure calculated from Hamiltonian (Suppl. 26) via general formalism [6] within wide-band limit is following:

$$T_D = \frac{4\Gamma^2\tau^{10}\tilde{\omega}^4}{Q_D + 4\Gamma^2\tau^{10}\tilde{\omega}^4}, \qquad \text{(Suppl. 31)}$$

with

$$Q_D = \tilde{\omega}^8 - 2\tilde{\varepsilon}_1\tilde{\omega}^7 + \left(\tilde{\varepsilon}_1^2 + \Gamma^2 - 8\tau^2\right)\tilde{\omega}^6 + 2\tilde{\varepsilon}_1\left(6\tau^2 - \Gamma^2\right)\tilde{\omega}^5 + \left[\tilde{\varepsilon}_1^2\Gamma^2 - \left(5\tilde{\varepsilon}_1^2 + 6\Gamma^2\right)\tau^2 + 16\tau^4\right]\tilde{\omega}^4$$
$$-2\tilde{\varepsilon}_1\tau^2\left(9\tau^2 - 4\Gamma^2\right)\tilde{\omega}^3 + \left[\left(6\tilde{\varepsilon}_1^2 + 5\Gamma^2\right)\tau^4 - 3\tilde{\varepsilon}_1^2\Gamma^2\tau^2 - 9\tau^6\right]\tilde{\omega}^2 + 2\tilde{\varepsilon}_1\tau^4\left(3\tau^2 - 2\Gamma^2\right)\tilde{\omega} - \tau^4\left[\tilde{\varepsilon}_1^2\left(\tau^2 - \Gamma^2\right) + \Gamma^2\tau^2\right]. \qquad \text{(Suppl. 32)}$$

Figure 6 shows the correspondence between exact transmission coefficient expression and estimation via our microscopic model with parameters $\gamma_{s,a}$ and $k_{s,a}$ obtained above. In contrast to the case of the non-disjoint diradical, here response of the transmission profile changes to the perturbation depends not only on the relation of perturbation to Γ but also to τ. This is due to the non-linear shift of the degenerate states energies, which suppresses the perturbation influence for low ratio δ/τ. All energies are measured in units of Γ.

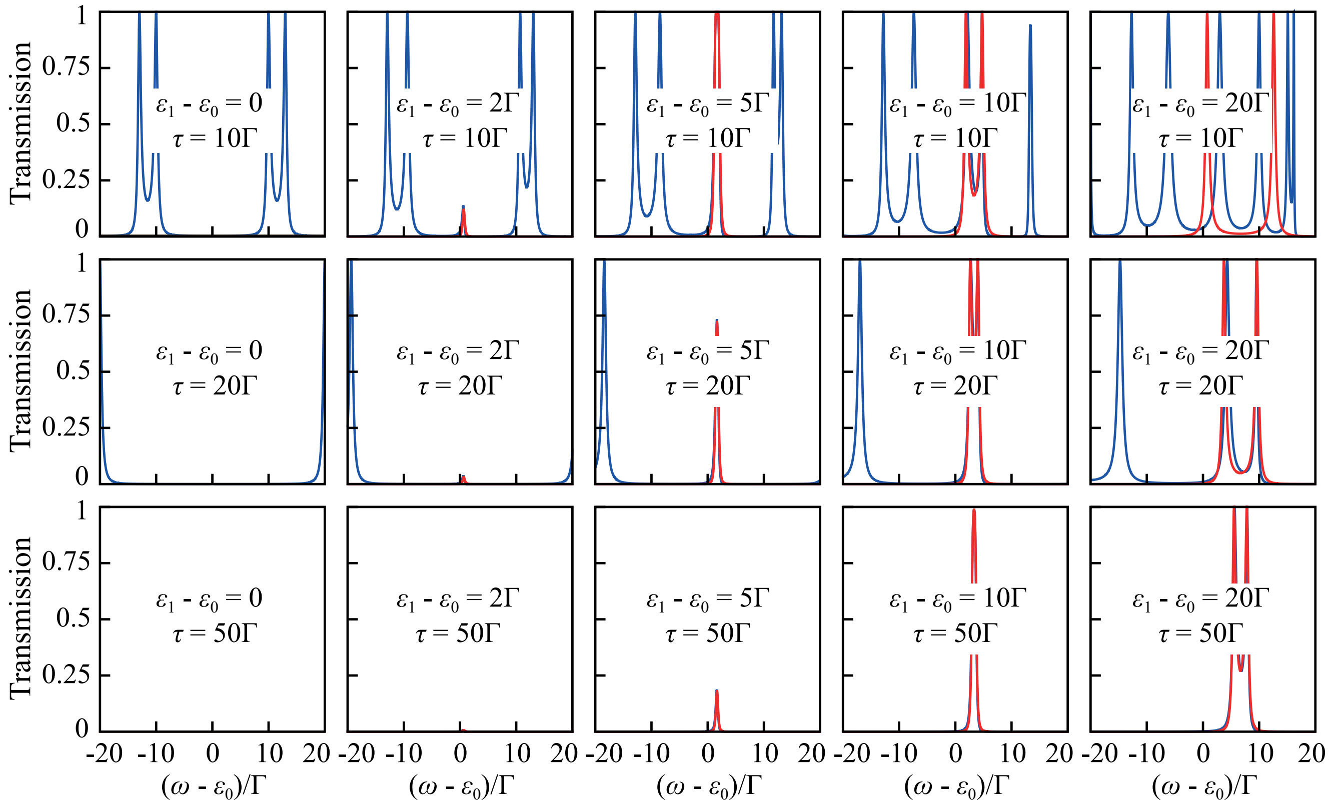

Suppl. Figure 6: Exact (blue lines) and estimated (red lines) transmission coefficient of a tight-binding Hückel model of divinylcyclobutadiene. In this case again the smaller couplings to the leads Γ are, the better agreement of the estimation by our microscopic model with exact solution becomes. Also these plots illustrate the fact that sensitivity of the transmission profile to the perturbation decreases as the perturbation strength becomes smaller compared to intermolecular hopping τ.

5 Few steps towards realistic description

5.1 Beyond wide-band limit

All the calculations of the transport properties (in the main text and above) are performed under the approximation of the leads with wide band, i.e. featureless contacts (with energy independent density of states and hopping integrals from the lead into the quantum conductor), which provide only imaginary part of the corresponding self-energy [12]. This approximation is used in many analytical calculations as it provides crucial simplification of the formulas derivation. It is assumed to give qualitative picture and it does not pretend to provide a quantitatively correct results. However, in some cases it can be in a good agreement with *ab initio* calculations [13]. For instance, it can be adequate in the case of gold contacts, which conduction band is about 8 eV and Fermi level is almost in its middle, whereas typical couplings (i.e. resonance width) are much smaller than 1 eV [14].

Nevertheless it is worth to consider the influence of the energy dependence of the contact self-energy on the transport properties of the proposed diradical quantum switches. We model contacts as 1D semi-infinite tight-binding linear chains of atoms with on-site potentials taken as energy origin and nearest-neighbour hoppings set as energy unity. Thus, contact self-energies in the basis of on-site localized states will be

$$\hat{\Sigma}_L(\omega) = \begin{pmatrix} \delta(\omega) - i\Gamma(\omega) & 0 & 0 & 0 \\ 0 & 0 & 0 & 0 \\ 0 & 0 & 0 & 0 \\ 0 & 0 & 0 & 0 \end{pmatrix}, \quad \hat{\Sigma}_R(\omega) = \begin{pmatrix} 0 & 0 & 0 & 0 \\ 0 & 0 & 0 & 0 \\ 0 & 0 & 0 & 0 \\ 0 & 0 & 0 & \delta(\omega) - i\Gamma(\omega) \end{pmatrix} \qquad \text{(Suppl. 33)}$$

for trimethylenemethane molecule and

$$\hat{\Sigma}_L(\omega) = \begin{pmatrix} 0 & 0 & 0 & 0 & 0 & 0 & 0 & 0 \\ 0 & 0 & 0 & 0 & 0 & 0 & 0 & 0 \\ 0 & 0 & 0 & 0 & 0 & 0 & 0 & 0 \\ 0 & 0 & 0 & 0 & 0 & 0 & 0 & 0 \\ 0 & 0 & 0 & 0 & 0 & 0 & 0 & 0 \\ 0 & 0 & 0 & 0 & 0 & 0 & 0 & 0 \\ 0 & 0 & 0 & 0 & 0 & 0 & \delta(\omega) - i\Gamma(\omega) & 0 \\ 0 & 0 & 0 & 0 & 0 & 0 & 0 & 0 \end{pmatrix}, \quad \hat{\Sigma}_R(\omega) = \begin{pmatrix} 0 & 0 & 0 & 0 & 0 & 0 & 0 & 0 \\ 0 & 0 & 0 & 0 & 0 & 0 & 0 & 0 \\ 0 & 0 & 0 & 0 & 0 & 0 & 0 & 0 \\ 0 & 0 & 0 & 0 & 0 & 0 & 0 & 0 \\ 0 & 0 & 0 & 0 & 0 & 0 & 0 & 0 \\ 0 & 0 & 0 & 0 & 0 & 0 & 0 & 0 \\ 0 & 0 & 0 & 0 & 0 & 0 & 0 & 0 \\ 0 & 0 & 0 & 0 & 0 & 0 & 0 & \delta(\omega) - i\Gamma(\omega) \end{pmatrix} \qquad \text{(Suppl. 34)}$$

for divinylcyclobutadiene molecule. Here $\delta(\omega)$ and $\Gamma(\omega)$ are given by

$$\delta(\omega) = \frac{\omega J^2}{2}, \quad \Gamma(\omega) = \frac{J^2}{2}\sqrt{4-\omega^2} \qquad \text{(Suppl. 35)}$$

with J being the tunneling matrix element between the first site of the contacts chain and the corresponding site of the molecule. Figure (7) depicts plots of the evolution of the transmission coefficient profile with varying energy ε_1 of externally controlled sites for different shifts of the overall molecule energy from the contact band center (where the wide-band limit works perfectly). Shifting the overall molecule energy from the band center destructively affects the switching properties of the non-disjoint diradical (trimethylenemethane) and near the band edge ($\varepsilon_0 = -1.5$) it completely loses its switching properties. On the other hand, the properties of the disjoint diradical (divinylcyclobutadiene) are immune to the shift from the band center and hence to the energy dependence of the self-energy (at least in this simple case).

5.2 Electrostatic influence (capacitive couplings) of contacts

Consider the electrostatic influence of the voltage applied between the left and the right contacts on the electron potential energy in the molecule as an example of imperfection, which breaks the mirror symmetry of initially symmetric system of molecule and the leads. Assume that voltage applied to the lead influences only the site this lead is connected to. So, for trimethylenemethane molecule we have the energy of the 1-st site (see Fig. 3a): $\varepsilon_1 = \varepsilon_0 + \alpha_L V_L$ and the energy of the 4-th site: $\varepsilon_4 = \varepsilon_0 + \alpha_R V_R$. For divinylcyclobutadiene we have (subscript corresponds to the site number in the scheme from Fig. 5a): $\varepsilon_7 = \varepsilon_0 + \alpha_L V_L$ and $\varepsilon_8 = \varepsilon_0 + \alpha_R V_R$. Figure 8 depicts current vs. gated sites energy ε_1 plots with different values of electrostatic lever arms $\alpha_L = \alpha_R = \alpha_c$ for disjoint and non-disjoint diradical-based quantum switches.

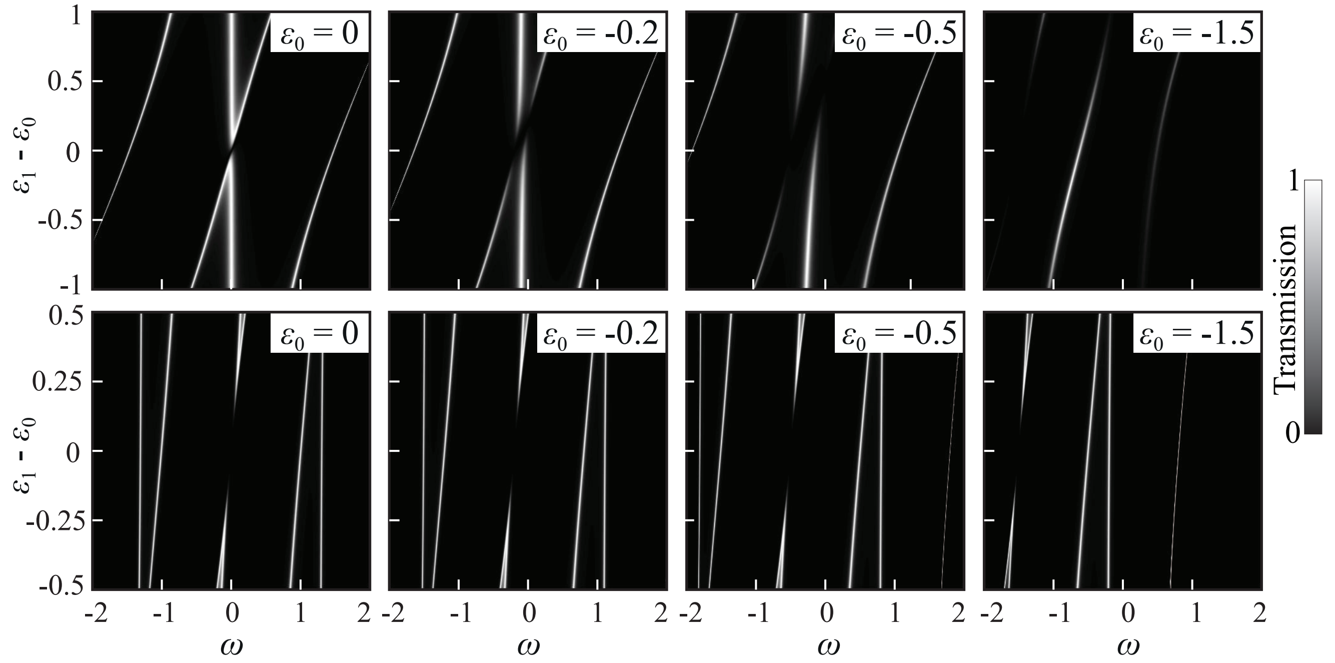

Suppl. Figure 7: Evolution of the transmission coefficient spectrum with varying energy ε_1 of externally controlled sites of trimethylenemethane molecule (the first row) and divinylcyclobutadiene molecule (the second row). Tight-binding parameters are chosen as follows: $\tau_1 = 0.75$, $\tau = 1$ and $J = 0.2$ for trimethylenemethane (τ/τ_1 corresponds to the typical value of the double/single bond hopping integrals ratio [15]) and $\tau = 1$, $J = 0.2$ for divinylcyclobutadiene. All energies are taken in units of the hopping integral in the leads.

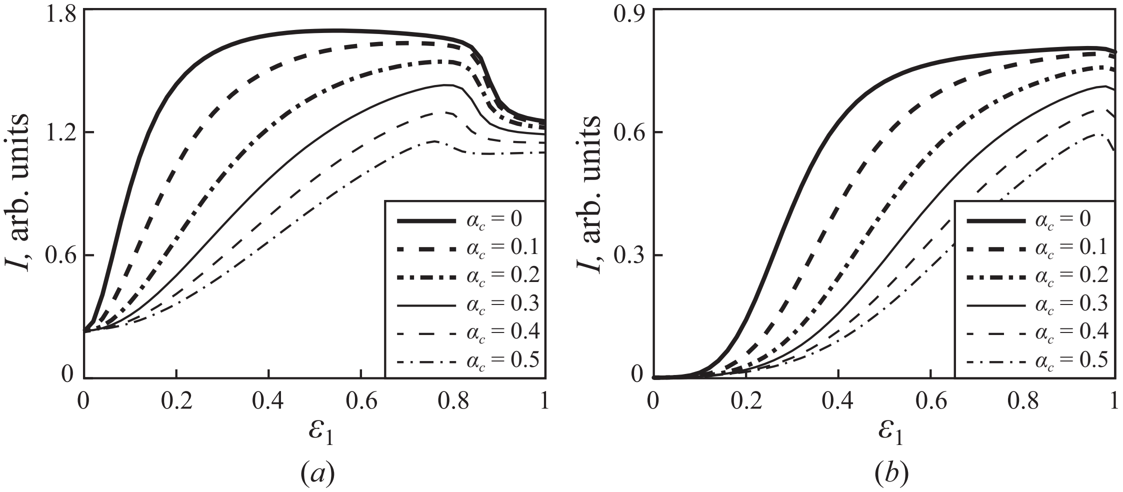

Suppl. Figure 8: Current vs. gated sites energy ε_1 plots for (a) non-disjoint and (b) disjoint diradical-based quantum switch. Voltage is applied symmetrically: $eV_L = -eV_0/2$ and $eV_R = eV_0/2$ with eV_0 set to 1. Leads are modeled as semi-infinite 1D chains and the energy ε_0 is set to the band center. Fermi energy of the unbiased leads is also set to the band center and temperature is zero. Tight-binding parameters are chosen as follows: $\tau_1 = 0.75$, $\tau = 1$ (ratio τ/τ_1 is typical for the double/single bond hopping integrals [15]) and $J = 0.2$ for trimethylenemethane (a) and $\tau = 1$, $J = 0.2$ for divinylcyclobutadiene (b). All energies are taken in units of the hopping integral in the leads.

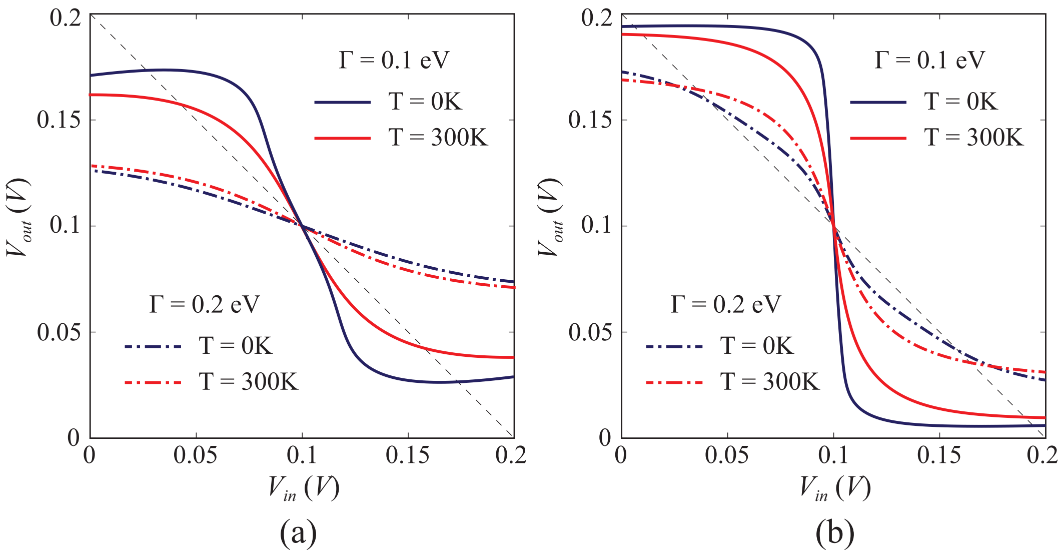

Suppl. Figure 9: Voltage transfer characteristics of the quantum interference inverter based on (a) non-disjoint diradical (trimethylenemethane) and (b) disjoint diradical (divinylcyclobutadiene) calculated for different temperature (blue lines – $T = 0$ and red lines – $T = 300$K) and couplings to the leads (solid lines – $\Gamma = 0.1$eV and dot-dashed lines – $\Gamma = 0.2$eV). Tight-binding parameters of trimethylenemethane and divinylcyclobutadiene are chosen as in the main text. Dashed black line shows the -1 slope for comparison.

From Fig. 8 one can see that electrostatic influence of the leads reduces the "on" state current. This is easily understood as such imperfections (which break the mirror symmetry) prevent the system from reaching the exact PT-symmetric regime with perfect tranmission peaks, thus, resulting in reduction of the transmission and, consequently, the current. For realistic values of the electrostatic lever arms of the current flowing leads (less than for a control gate contact, e.g., $\alpha = 0.5$ taken in the calculations in the main text), current reduction is not critical. Disjoint diradical shows a little more stability under this perturbation. Also one can admit that the I_{on}/I_{off} ratio does not suffer drastically from this electrostatic influence, especially, again, for the disjoint diradical switch. Current decreases for high shift ε_1 from the band center, because resonance goes out of a sharp integration range given by occupation difference in the leads at zero temperature.

5.3 Strong coupling to leads

Under the condition of the weak coupling to the leads, which was assumed in the main text ($\Gamma = 1$meV), electron-electron repulsion can provide a Coulomb blockade regime to build up. This effect will prevent current flowing for low voltages, therefore ruining the quantum switch performance. Coulomb repulsion must be properly described by *ab initio* calculations because of contact capacitance and many-bode electron correlations must be taken into account. However, it becomes less pronounced for stronger couplings to the leads, which lowers the contact resistance. Thus, one can increase Γ to decrease the influence of electron repulsion. On the other hand, high values of Γ will increase the influence of distant resonance peaks, which always exist in real molecules. Therefore, one should increase the supply voltage to retain the switching properties of the quantum interference device. Here we provide voltage transfer characteristics of the quantum interference inverter (Fig. 9) calculated within the wide-band approximation for higher operating voltage $V_0 = 0.2$V and much stronger couplings to the leads $\Gamma = 0.1$eV and $\Gamma = 0.2$eV compared to those in the main text. One can see that disjoint diradical based device again shows better characteristics (with higher gain).

6 Formalism of P and Q functions within source-sink potential (SSP) approach

Proposed in Ref. [16] the source-sink potential (SSP) method provides a description of transport properties of molecular systems, by incorporating scattering boundary conditions into the complex self-energies. This was also independently established in Refs. [17, 18], where connection to $\mathcal{PT}$-symmetry was also highlighted. The SSP approach allows for a simple formula for the transmission coefficient of a molecular conductor, at least within the Hückel description via bare tight-binding Hamiltonian $\hat{H}_0$ and under the assumption that the leads are attached to only one atom each (with some indexes r and s) [19, 20]:

$$T = \frac{4\beta_L\beta_R\left(\Delta_{r,r}\Delta_{s,s} - \Delta\Delta_{rs,rs}\right)}{\left|\Delta + i\beta_L\delta_{s,s} + i\beta_R\Delta_{r,r} - \beta_L\beta_R\Delta_{rs,rs}\right|^2}, \qquad \text{(Suppl. 36)}$$

where $\Delta = \det(\omega\hat{I} - \hat{H}_0)$ and $\Delta_{i,j}$ denotes to the minor of $(\omega\hat{I} - \hat{H}_0)$ with i-th row and j-th column crossed out. Quantities $\beta_{L,R}$ describe couplings with the leads. As was noted in Ref. [19], according to the Jacobi theorem [21], the numerator of this formula is proportional to the square of the minor $\Delta_{r,s}$: $4\beta_L\beta_R(\Delta_{r,r}\Delta_{s,s} - \Delta\Delta_{rs,rs}) = |2\sqrt{\beta_L\beta_R}\Delta_{r,s}|^2$, which exactly coincides with the expression for the function P for this case [6]. Thus, the transmission coefficient (Suppl. 36) can be written in the form $T = |P|^2(|P|^2 + |Q|^2)^{-1}$, with

$$\begin{aligned} P &= 2\sqrt{\beta_L\beta_R}\Delta_{r,s}, \\ Q &= \Delta - i\beta_L\Delta_{s,s} + i\beta_R\Delta_{r,r} + \beta_L\beta_R\Delta_{rs,rs}. \end{aligned} \qquad \text{(Suppl. 37)}$$

One can easily see that Q from (Suppl. 37) is a characteristic polynomial of the bare Hamiltonian of the system $\hat{H}_0$ with additional terms $-i\beta_L$ and $+i\beta_R$ in the (s,s) and (r,r) positions correspondingly. In other words, Q is nothing but a characteristic polynomial of the auxiliary Hamiltonian of the system. Real roots of Q define perfect transmission energies, in particular, for even alternant hydrocarbons at zero energy (energy of carbon p-orbital) $\Delta_{r,r}(0) = \Delta_{s,s}(0) = 0$ and the perfect transmission condition ($Q = 0$) is reduced to $(\beta_L\beta_R)^{-1} = -\frac{\Delta_{rs,rs}}{\Delta} = T^{rel}_{r,s}$, which was established in Ref. [20]. Here $T^{rel}_{r,s}$ is a "relative transmission" defined in [20].

References

[1] Carl M. Bender and Stefan Boettcher. Real spectra in non-hermitian hamiltonians having PT symmetry. *Phys. Rev. Lett.*, 80:5243–5246, Jun 1998.

[2] Carl M Bender. Making sense of non-hermitian hamiltonians. *Reports on Progress in Physics*, 70(6):947, 2007.

[3] Carl M Bender. Introduction to -symmetric quantum theory. *Contemporary Physics*, 46(4):277–292, 2005.

[4] Ramy El-Ganainy, Konstantinos G. Makris, Mercedeh Khajavikhan, Ziad H. Musslimani, Stefan Rotter, and Demetrios N. Christodoulides. Non-hermitian physics and pt symmetry. *Nature Physics*, 14:11, Jan 2018. Review Article.

[5] T. Kato. *Perturbation Theory for Linear Operators.* Classics in Mathematics. Springer-Verlag, Berlin, Heidelberg, 1995.

[6] A. A. Gorbatsevich and N. M. Shubin. Unified theory of resonances and bound states in the continuum in hermitian tight-binding models. *Phys. Rev. B*, 96:205441, Nov 2017.

[7] Lev Davidovich Landau and Evgenii Mikhailovich Lifshitz. *Quantum mechanics: non-relativistic theory*, volume 3. Elsevier, Oxford, 2013.

[8] C Caroli, R Combescot, P Nozieres, and D Saint-James. Direct calculation of the tunneling current. *Journal of Physics C: Solid State Physics*, 4(8):916, 1971.

[9] Yigal Meir and Ned S. Wingreen. Landauer formula for the current through an interacting electron region. *Phys. Rev. Lett.*, 68:2512–2515, Apr 1992.

[10] S. Datta. *Electronic Transport in Mesoscopic Systems*. Cambridge Studies in Semiconductor Physics. Cambridge University Press, 1997.

[11] Chenming Hu. *Modern semiconductor devices for integrated circuits*, volume 1. Prentice Hall Upper Saddle River, NJ, 2010.

[12] D.A. Ryndyk, R Gutiérrez, B Song, and G Cuniberti. Green function techniques in the treatment of quantum transport at the molecular scale. In *Energy Transfer Dynamics in Biomaterial Systems*, pages 213–335. Springer, Berlin, Heidelberg, 2009.

[13] C. J. O. Verzijl, J. S. Seldenthuis, and J. M. Thijssen. Applicability of the wide-band limit in dft-based molecular transport calculations. *The Journal of Chemical Physics*, 138(9):094102, 2013.

[14] Matías Zilly. *Electronic conduction in linear quantum systems: Coherent transport and the effects of decoherence*. PhD thesis, Universität Duisburg-Essen, Fakultät für Physik» Theoretische Physik, 2010.

[15] A. J. Heeger, S. Kivelson, J. R. Schrieffer, and W. P. Su. Solitons in conducting polymers. *Rev. Mod. Phys.*, 60:781–850, Jul 1988.

[16] Francois Goyer, Matthias Ernzerhof, and Min Zhuang. Source and sink potentials for the description of open systems with a stationary current passing through. *The Journal of Chemical Physics*, 126(14):144104, 2007.

[17] L. Jin and Z. Song. Physics counterpart of the $\mathcal{PT}$ non-hermitian tight-binding chain. *Phys. Rev. A*, 81:032109, Mar 2010.

[18] L Jin and Z Song. A physical interpretation for the non-hermitian hamiltonian. *Journal of Physics A: Mathematical and Theoretical*, 44(37):375304, 2011.

[19] B.T. Pickup and P.W. Fowler. An analytical model for steady-state currents in conjugated systems. *Chemical Physics Letters*, 459(1):198 – 202, 2008.

[20] Thijs Stuyver, Stijn Fias, Frank De Proft, Paul Geerlings, Yuta Tsuji, and Roald Hoffmann. Enhancing the conductivity of molecular electronic devices. *The Journal of Chemical Physics*, 146(9):092310, 2017.

[21] I.S. Gradshteyn and I.M. Ryzhik. *Table of Integrals, Series, and Products*. Elsevier Science, 2014.
